# Spread of *Botrytis cinerea* Strains with Multiple Fungicide Resistance in German Horticulture

**DOI:** 10.3389/fmicb.2016.02075

**Published:** 2017-01-03

**Authors:** Sabrina Rupp, Roland W. S. Weber, Daniel Rieger, Peter Detzel, Matthias Hahn

**Affiliations:** ^1^Department of Biology, University of KaiserslauternKaiserslautern, Germany; ^2^Esteburg Fruit Research and Advisory CentreJork, Germany; ^3^Department of Food Science, Aarhus UniversityÅrslev, Denmark; ^4^Belchim Crop ProtectionBurgdorf, Germany; ^5^NüPA GmbHKarlsruhe, Germany

**Keywords:** *Botrytis cinerea*, fitness, fungicide resistance, Germany, multiple resistance, ornamental flowers, raspberry, strawberry

## Abstract

*Botrytis cinerea* is a major plant pathogen, causing gray mold rot in a variety of cultures. Repeated fungicide applications are common but have resulted in the development of fungal populations with resistance to one or more fungicides. In this study, we have monitored fungicide resistance frequencies and the occurrence of multiple resistance in *Botrytis* isolates from raspberries, strawberries, grapes, stone fruits and ornamental flowers in Germany in 2010 to 2015. High frequencies of resistance to all classes of botryticides was common in all cultures, and isolates with multiple fungicide resistance represented a major part of the populations. A monitoring in a raspberry field over six seasons revealed a continuous increase in resistance frequencies and the emergence of multiresistant *Botrytis* strains. In a cherry orchard and a vineyard, evidence of the immigration of multiresistant strains from the outside was obtained. Inoculation experiments with fungicide-treated leaves in the laboratory and with strawberry plants cultivated in the greenhouse or outdoors revealed a nearly complete loss of fungicide efficacy against multiresistant strains. *B. cinerea* field strains carrying multiple resistance mutations against all classes of site-specific fungicides were found to show similar fitness as sensitive field strains under laboratory conditions, based on their vegetative growth, reproduction, stress resistance, virulence and competitiveness in mixed infection experiments. Our data indicate an alarming increase in the occurrence of multiresistance in *B. cinerea* populations from different cultures, which presents a major threat to the chemical control of gray mold.

## Introduction

Gray mold is a destructive fungal disease of a wide range of fruit, vegetable and ornamental crops. Infections are usually initiated by conidia. They may or may not enter a state of latency before an aggressive necrotrophic rot breaks out, soon followed by the production of a dense gray lawn of new conidia. Formerly thought to be caused by one species called *Botrytis cinerea* Pers.:Fr., gray mold is now known to be due to a complex of cryptic species (Walker et al., 2011; Plesken et al., 2015a). In addition, the core species of *B. cinerea* can be separated into genetically more or less distinct groups (N and S) which were shown to be enriched differentially in response to fungicide treatments (Leroch et al., 2013; Plesken et al., 2015a).

In many horticultural crops, gray mold is the most serious of all fungal diseases, and repeated fungicide sprays are necessary to control the pathogen especially in mild and humid climates. Due to the lack of registered broad-spectrum compounds, chemical control of *Botrytis* spp. almost solely relies on fungicides with specific modes of action. In German strawberry production, which is representative of many other European countries, the following fungicide groups are in current use against *Botrytis*: Quinone-outside inhibitors (QoI) such as azoxystrobin, trifloxystrobin or pyraclostrobin; succinate dehydrogenase inhibitors (SDHI) such as boscalid or the recently registered fluopyram; anilinopyrimidines (e.g., cyprodinil, pyrimethanil, mepanipyrim); phenylpyrroles (fludioxonil); and hydroxyanilides (fenhexamid). Other fungicide groups such as dicarboximides (iprodione) or methyl benzimidazole carbamates (benomyl, thiophanate-methyl) have been discontinued in 2009 or the mid-1970s, respectively, although iprodione continues to be used in several vegetable and ornamental crops. The above mentioned fungicides are used against *Botrytis* at variable frequencies in different crops. Strawberries and raspberries are among the most intensively treated crops. Resistance to individual botryticides has been reported in Germany and other countries since the 1970s, affecting all major fungicide classes (Leroux et al., 2002; Hahn, 2014). In the past 5 years, increasing frequencies of *Botrytis* strains with multiple fungicide resistance (MR strains) have been reported in different parts of the world especially from strawberries (Amiri et al., 2013; Fernández-Ortuño et al., 2015), but also from raspberries (Weber, 2011; Plesken et al., 2015a), grapes (De Miccolis Angelini et al., 2014; Panebianco et al., 2015) and tomatoes (Konstantinou et al., 2015).

In this study, we have traced the development of fungicide resistance in *Botrytis* isolates obtained in 2010 to 2015 from different host plants in Germany that have been regularly treated with fungicides. In addition to high average resistance frequencies, we found increasing proportions over time of isolates with simultaneous resistance to several or even all fungicides. These MR strains failed to be controlled by any of the fungicides used against them in detached-leaf and potted-plant experiments. Genetic and phenotypic analyses of *B. cinerea* MR strains indicated that they are not significantly impaired in their growth, reproduction and infection performance compared to sensitive field strains. The relevance of these results to practical horticultural production is discussed.

## Materials and methods

### Isolation and cultivation of *Botrytis* strains

*Botrytis* strains were isolated from infected plant tissue by streaking out conidia onto potato dextrose agar (PDA) augmented with 200 mg penicillin G and 200 mg streptomycin sulfate L^−1^ (Weber and Hahn, 2011) or HA agar (10 g malt extract, 4 g glucose, 4 g yeast extract, 15 g agar per liter, pH 5.5) augmented with 5 mg tannic acid L^−1^, to inhibit growth of several contaminating fungi. Purified strains were cultivated on HA, and subjected to long-term storage by freezing to −80°C.

Growth tests of *B. cinerea* strains were performed on agar plates containing either HA or Gamborg GB5 minimal medium containing 25 mM glucose as described by Plesken et al. (2015b). For determination of radial growth, three 10 μL droplets containing 10^5^ conidia mL^−1^ were applied to the center of a 9 cm agar plate, and incubated for 72 h before evaluation of the colony diameter.

### Fungicide sensitivity assays

Fungicide sensitivity assays were performed by analysing germination and growth of conidia on agar plates containing discriminatory fungicide concentrations. Because these assays were started in 2010 and performed in two different labs, prior to the decision for a joint publication, two fungicide resistance assays were used. The method of Weber and Hahn (2011) was used for *Botrytis* isolates collected in Northern Germany, whereas all other isolates were examined according to Leroch et al. (2013). Quantitative evaluation of fungicide resistance levels was performed as described by Leroch et al. (2013). Active ingredients were obtained by using single-compound fungicides, as follows: fenhexamid from Teldor (Bayer CropScience, Monheim, Germany); trifloxystrobin from Flint (Bayer) or azoxystrobin from Ortiva (Syngenta, Basel, Switzerland); boscalid from Cantus (BASF, Limburgerhof, Germany); cyprodinil from Chorus (Syngenta); fludioxonil from Saphire (Syngenta); iprodione from Rovral WG (BASF) and thiophanate-methyl from Cercobin FL (BASF). The SDHI compound fluopyram was also sometimes tested but data are not reported here because this compound was not registered until the 2015 season (Weber et al., 2015).

For *in planta* assays, commercial fungicides were prepared as stock solutions in EtOH (1 mg mL^−1^) and adjusted to the recommended doses in water. For inoculation experiments of freshly detached tomato leaves, these were sprayed with fungicides (or water in the controls) to leaf wetness, and dried in a sterile air flow cabinet. After inoculation with 20 μl droplets of spore suspension (10^5^ conidia mL^−1^ in GB5 medium with 25 mM glucose), leaves were incubated in a moist chamber and checked for lesion development after 72 h.

For the greenhouse and outdoor inoculation experiments, strawberry plants (cv. Sonata) were raised in pots (9 cm diameter) filled with TKS1 peat-based substrate (Floragard, Oldenburg, Germany). The pots were placed in trays and watered by drop irrigation. After 6 weeks, plants carrying immature fruits were treated twice at an interval of 7–9 days with ~5 mL fungicide solution per plant using a hand-held spray bottle, each time followed 24 h later by spray inoculation with 1 mL of *B. cinerea* spore suspension (3 × 10^5^ conidia mL^−1^) per plant. Fungicide applications were conducted with Teldor® (2 g L^−1^; active ingredient fenhexamid), Signum® (1.8 g L^−1^; boscalid + pyraclostrobin) or Switch® (1 g L^−1^; cyprodinil + fludioxonil). Inoculation was performed with the fully sensitive *B. cinerea* strain B05.10 or the multiresistant strain MR-S4 (**Table 2**). Each experimental variant including unsprayed inoculated and unsprayed uninoculated controls comprised six plants placed at random. Fruits were harvested 5–17 days after the first inoculation. Rotting fruit were collected at harvest and again after incubation in a humid chamber for up to 1 week. From sporulating lesions of all infected fruits, conidia were recovered and tested on agar plates with discriminatory fungicide concentrations for their resistance against fenhexamid, a QoI fungicide (azoxystrobin or trifloxystrobin), boscalid, cyprodinil and fludioxonil. Isolates with resistance patterns that differed from the inoculated strain were considered as immigrated contaminant strains, and excluded from evaluation of the specific infection rate.

### Fitness assays

Competitive infection assays with apples were performed as follows: Apples (cv. Golden Delicious) were surface-sterilized by submersion for 5 min in a 1% sodium hypochlorite solution, rinsed three times with sterile water, and dried in a sterile air-flow cabinet. Fruits were wounded at two or four equidistant sites above the fruit equator by removing the peel with a sterile 6 mm diameter stainless steel cork borer. Wounds were inoculated with 20 μL of a conidial suspension of *B. cinerea*. The suspension was prepared by adjusting conidia suspended in GB5 medium containing 25 mM glucose to a concentration of 2 × 10^5^ mL^−1^, and mixing either with the suspension of another *B. cinerea* strain or with medium alone (controls). Inoculated apples were incubated in moist plastic boxes for 7 days. Subsequently, the fruit was divided into two or four pieces, and from each side a tangential cut was made in order to expose the rotten area to the air and to promote sporulation. Spores were then collected and the spore suspension was transferred onto HA agar plates supplemented either with 10 ppm fenhexamid to distinguish between sensitive and MR strains, or with 0.2 ppm or 1 ppm fludioxonil to distinguish between strains with or without partial fludioxonil resistance due to the MDR1(h) efflux mechanism.

Sclerotium formation was induced by incubation of HA plates with mycelium-containing agar plaques for 4 weeks at 15°C in the dark. Viability of sclerotia was tested by storing the plates for 8 months at 10–14°C, followed by transfer of five individual sclerotia per strain onto a fresh HA agar plate and incubation at room temperature. After 3 days, development of mycelium from the sclerotia was checked.

### DNA manipulations

For determination of fungicide resistance mutations of selected *B. cinerea* isolates, total DNA was isolated as described by Leroch et al. (2013). Mutations were identified by amplification of target gene fragments, followed by either sequencing or primer-induced restriction analysis (PIRA)-PCR (Haliassos et al., 1989) to identify specific mutations. The F412S mutations in *erg27*, the G143A mutation in *cytB*, the H272R and the H272Y mutations in *sdhB*, the E198A mutation in *tubA*, and the Δ*L497* mutation in *mrr1* were identified by PIRA-PCR, using the primers and enzymes shown in Table 1. Restriction enzymes were purchased from NEB Biolabs (Frankfurt, Germany). Mutations in *bos1* conferring iprodione resistance were identified by pyrosequencing (Grabke and Stammler, 2015). All other mutations were identified by sequencing.

**Table 1:** **Primers used in this study**.

**Name**	**Sequence (5′–3′)**	**Amplicon length**	Gene (mutation^*^)	**References**
BpsID_137F	GCAGATGAGGCGGATGATAG	137/112 bp^**^	BC1G_07159/Bcin09g02270	Plesken et al., 2015a
BpsID_273R	TCCACCCAAGCATCATCTTC			
BcinN-in-F	GCGACCTCATCGTTCTTTCAC	182 bp	*mrr1*	Plesken et al., 2015a
BcinN-in-R	GGCTCTCGATGAGCTGTTTC			
Mrr1-spez-F	TATCGGTCTTGCAGTCCGC	141 bp	*mrr1*	Leroch et al., 2013
Mrr1-spez-R	TTCCGTACCCCGATCTTCGGAA			
erg27F412SP745F	GTGTTGGTATTTATCTACAGATTGATAT	125 bp	*erg27* (F412S^*1^)	This work
erg27_1410R	ACATCGTCGGGAGATAATGC			
Seq_erg27_fw	GTACCGCCACTTATTCCG	950 bp	*erg27* (F412I)	This work
Seq_erg27_rev	GCGTTCTTTCCCACTAGC			
Seq_erg27_I63	CAGGCGCAAACAGGTGATAC	1425 bp	*erg27* (T63I)	This work
Seq_erg27_rev	GCGTTCTTTCCCACTAGC			
Qo13ext	GGTATAACCCGACGGGGTTATAGAATAG	560 bp^***^	*cytB* (G143A^*2^)	Samuel et al., 2011
Qo14ext	AACCATCTCCATCCACCATACCTACAAA			
H272R-fw	GGCAGCTTTGGATAACAGCATGAGTTTGTACAGATGGC	120 bp	*sdhB* (H272R^*3^)	Veloukas et al., 2011
H272-univ-rev	GCCATTTCCTTCTTAATCTCCGC			
H272Y-fwfw	GGCAGCTTTGGATAACAGCATGAGTTTGTACAGATAT	120 bp	*sdhB* (H272Y^*4^)	Veloukas et al., 2011
H272-univ-rev	GCCATTTCCTTCTTAATCTCCGC			
Mrr1_spez_F	TATCGGTCTTGCAGTCCGC	442 bp	*mrr1* (ΔL497^*5^)	Leroch et al., 2013
Mrr1-Pira	CCACCACAATCTTGGATCATTGGGATCAGAACCTGC			
PIRA-Tub1	GCCTCGTTATCGATACAGAAGATC	132 bp	*tubA* (E198A^*6^)	Plesken et al., 2015a
PIRA-Tub2	TATGATGGCTACCTTCTCCGTC			
BF2	CAACGTTATGGCACAAAATCTCA	849 bp	*bos1*	Ma et al., 2007
BR2	AAGTTTCTGGCCATGGTGTTCA			

* *Fungicide resistance mutation detected*.

** *Amplicon sizes for B. cinerea and B. pseudocinerea, respectively*.

*** *Amplicon size if no intron is present*.

*The following enzymes were used for digestion of the PCR fragments to detect the indicated mutation*:

*1 *EcoRV*;

*2 *SatI*;

*3 *HhaI*;

*4 *EcoRV*;

*5 *HpyCH4V*;

*6 *BglII*.

### Statistics

For statistical analysis of the results, a two-sided unpaired *t*-test was performed (Prism 5 for Windows, Version 5.01).

## Results

### Multiple fungicide resistance in *Botrytis* strains from different cultures

Gray mold isolates from a variety of host plants were collected and analyzed for their sensitivity or resistance to the main classes of site-specific fungicides with activity against *Botrytis*. Our assays permitted the detection of medium and/or high resistance to all fungicides tested, including the five registered fungicide classes QoI (azoxystrobin or trifloxystrobin), boscalid, fenhexamid, cyprodinil and fludioxonil. In the following, strains with medium and high resistance were rated as resistant. Strains simultaneously resistant to four or all five registered fungicides are referred to as MR (multiresistant) strains.

**Figure 1:**
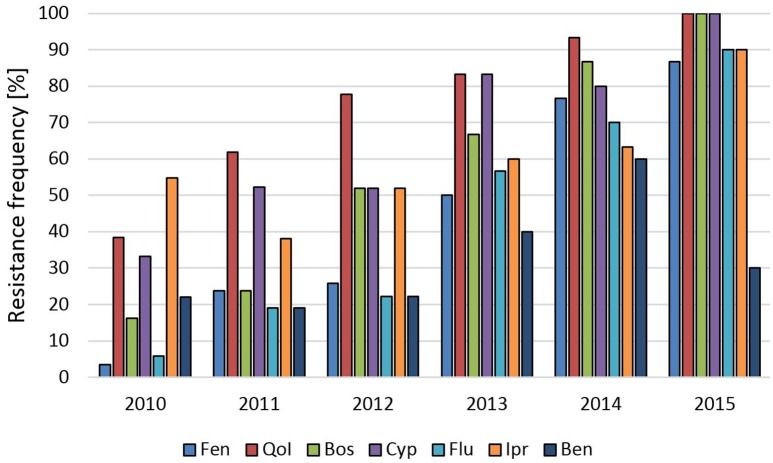
**Changes in frequencies of resistance of ***B. cinerea*** isolates to seven fungicides over 6 years in a raspberry field in Northern Germany**. Fen, fenhexamid; QoI, trifloxystrobin; Bos, boscalid; Cyp, cyprodinil; Flu, fludioxonil; Ipr, iprodione; Ben, thiophanate-methyl.

**Figure 2:**
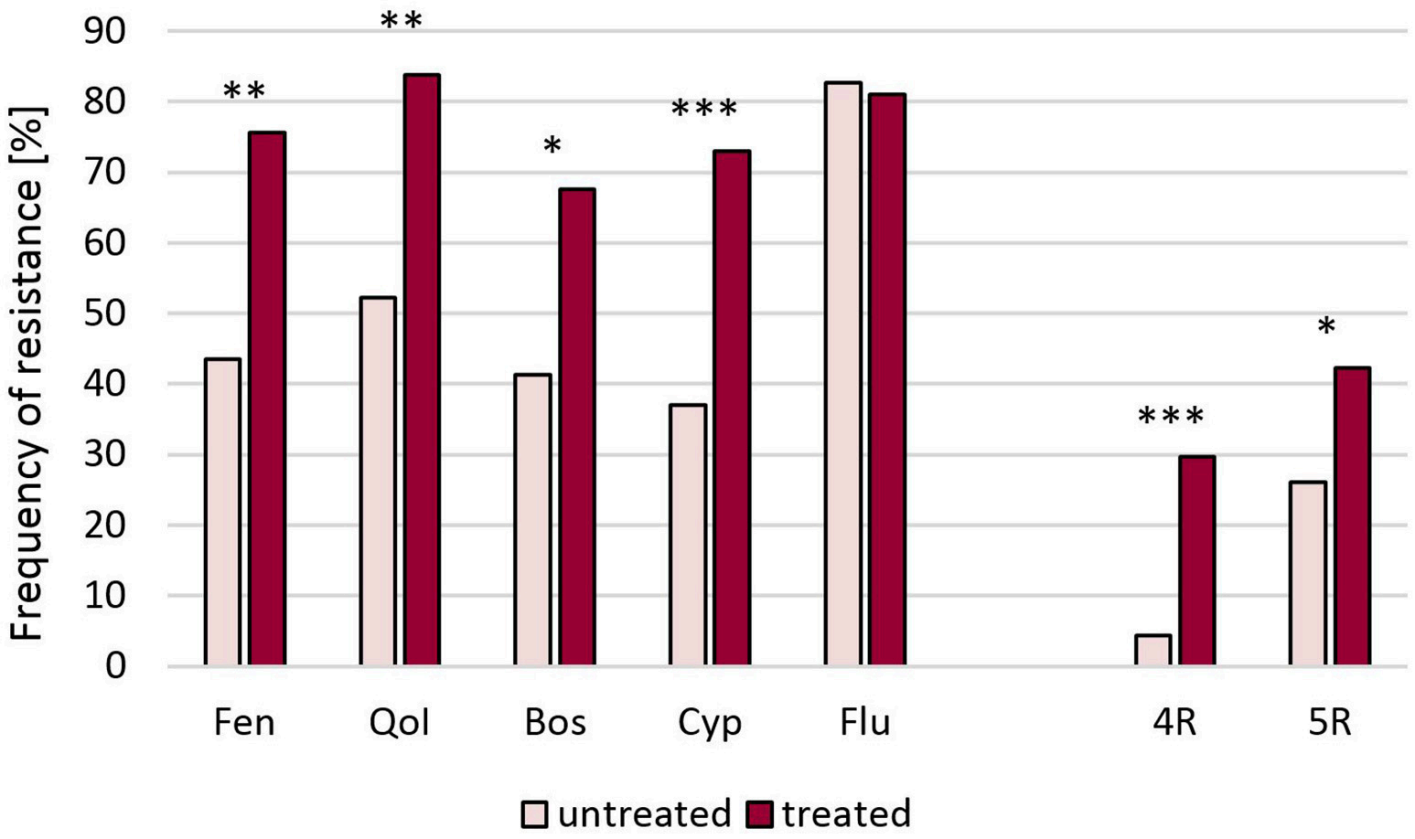
**Resistance frequencies of ***B. cinerea*** isolates from a field of everbearing strawberries in Southern Germany (2014) after eight fungicide treatments, and an untreated control plot**. Significant differences are indicated at ^*^p < 0.05, ^**^p < 0.01, and ^***^p < 0.001. Fen, fenhexamid; QoI, azoxystrobin; Bos, boscalid; Cyp, cyprodinil; Flu, fludioxonil. 4R, 5R, Strains with four or five resistances, respectively.

**Raspberries** receive repeated fungicide sprays against *Botrytis* during flowering. Plants are kept either as annual crops or cultivated for 10 years or more, thereby permitting long-term surveys of fungicide resistance development. One such field was planted in 2002 and examined in 2010–2015. In 2010 resistance frequencies were below 10% for fenhexamid and fludioxonil and between 15 and 55% for the other fungicides. In the following years, resistance frequencies steadily increased to more than 85% for all fungicides except for benzimidazoles, resulting in an almost completely resistant population (Figure 1). In line with this development, strains with 5-fold resistance to all registered fungicide classes were first recorded at harvest 2012 when they comprised 15% of the population, and their share rose dramatically to 87% in 2015 (data not shown). Similar trends were seen in two other raspberry fields (Table S1). **Strawberries** are grown in Germany in different cultivation forms for 2 to 3 years. Frequent fungicide treatments are carried out during the flowering period, and high resistance frequencies are common in these fields (Weber, 2011; Leroch et al., 2013). The data shown here are from isolates obtained in the 2013 seasons in six fields in Western and Southern Germany, which had all received four or more sprays with site-specific fungicides. Resistance frequencies approached 100% for all five classes of current *Botrytis* fungicides (Table S1). Everbearing strawberries with an extended harvest period from mid-summer to autumn are increasingly being cultivated in Germany for local or regional sale. These cultures are usually sprayed weekly against gray mold, raising an enormous selection pressure on the pathogen. *Botrytis* isolates from a field with everbearing strawberries in Southern Germany in 2014, treated for 8 weeks alternately with cyprodinil + fludioxonil or with fenhexamid, showed very high resistance frequencies. In an untreated plot within the same field, somewhat lower resistance frequencies were observed (Figure 2). Isolates from other fields of everbearing strawberries in Northern Germany also showed very high resistance levels, a high proportion of strains possessing 5-fold resistance to all fungicides registered against *Botrytis* (Table S1).

**Figure 3:**
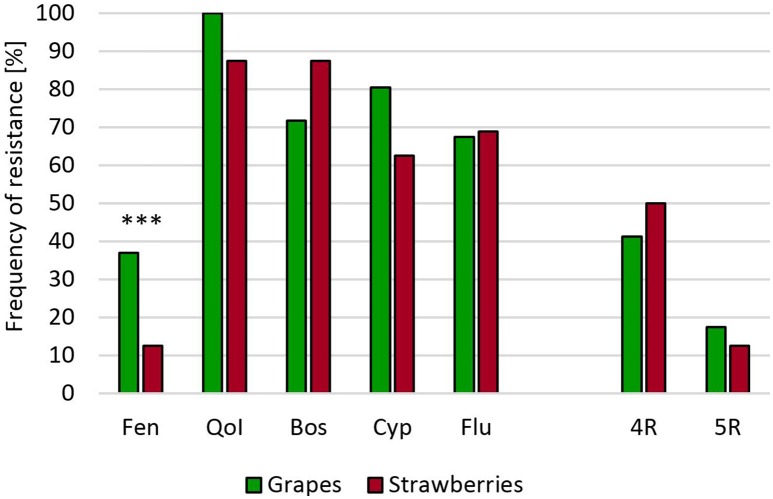
**Resistance frequencies of ***B. cinerea*** isolates from a vineyard and a neighboring strawberry field in Southern Germany (2014)**. Abbreviations are as in Figure 2. ^***^p < 0.001.

In the Wine Road Region of Germany, gray mold isolates from **grapevine berries** have been previously found to show moderate resistance frequencies to the registered fungicides (Leroch et al., 2011). A survey of isolates harvested in 2011 and 2014 revealed that resistance mainly occurred against QoI, whereas no or rather low levels of resistance existed against the other registered fungicides. Remarkably, the proportion of boscalid resistant isolates had increased from 4 to 35% between 2001 and 2014, probably due to the increased use of this fungicide (Table S1). None of the grapevine isolates had accumulated resistance to more than three registered fungicides. To test whether a transfer of MR strains from heavily treated strawberry fields into vineyards could occur, we analyzed a vineyard and an adjacent strawberry field in a fruit growing region in Southern Germany. Resistance frequencies in the strawberry and the grapevine populations were similar and much higher than resistance frequencies observed in the German Wine Road region (Figure 3).

**Figure 4:**
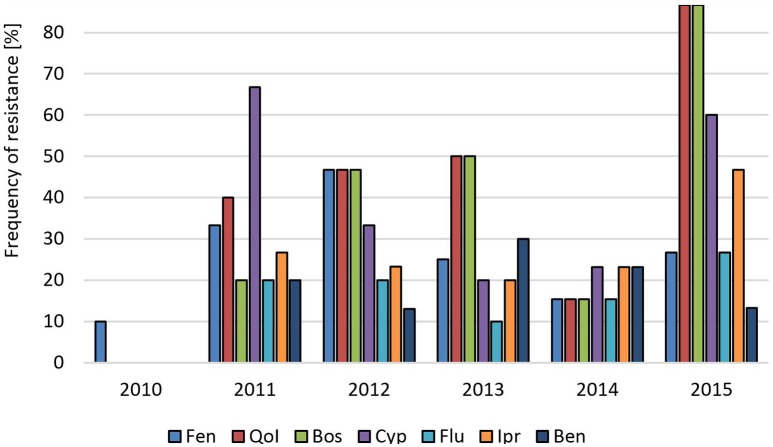
**Changes in fungicide resistance frequencies in ***B. cinerea*** isolates over 6 years in a sweet cherry orchard in Northern Germany**. Abbreviations are as in Figure 1.

In Germany, gray mold on **stone fruit** such as cherries and plums is less important than brown rot caused by *Monilinia* spp., and fungicide sprays are less frequent than in soft fruit fields. A long-term survey of a commercial cherry orchard conducted for 6 consecutive years revealed almost no resistance of any of the tested isolates in 2010. In 2011, a sudden increase in resistant isolates and the appearance of MR isolates was observed, which suggested their immigration from another field (Figure 4). The elevated resistance levels were largely maintained during subsequent years except for a transient decrease in 2014. Isolates obtained from four other cherry orchards in 2012, 2013, and 2014 showed intermediate to high resistance levels, and between 7 and 33% of strains were resistant to all five registered fungicides (Table S1). Isolates from a plum orchard in the same region sampled in 2012 showed high resistance frequencies, with 40% of all isolates being 5-fold resistant (Table S1). We also obtained a low number of isolates from tree seedlings raised in a tree nursery in Northern Germany, all of which belonged to the MR type (Table S1).

**Figure 5:**
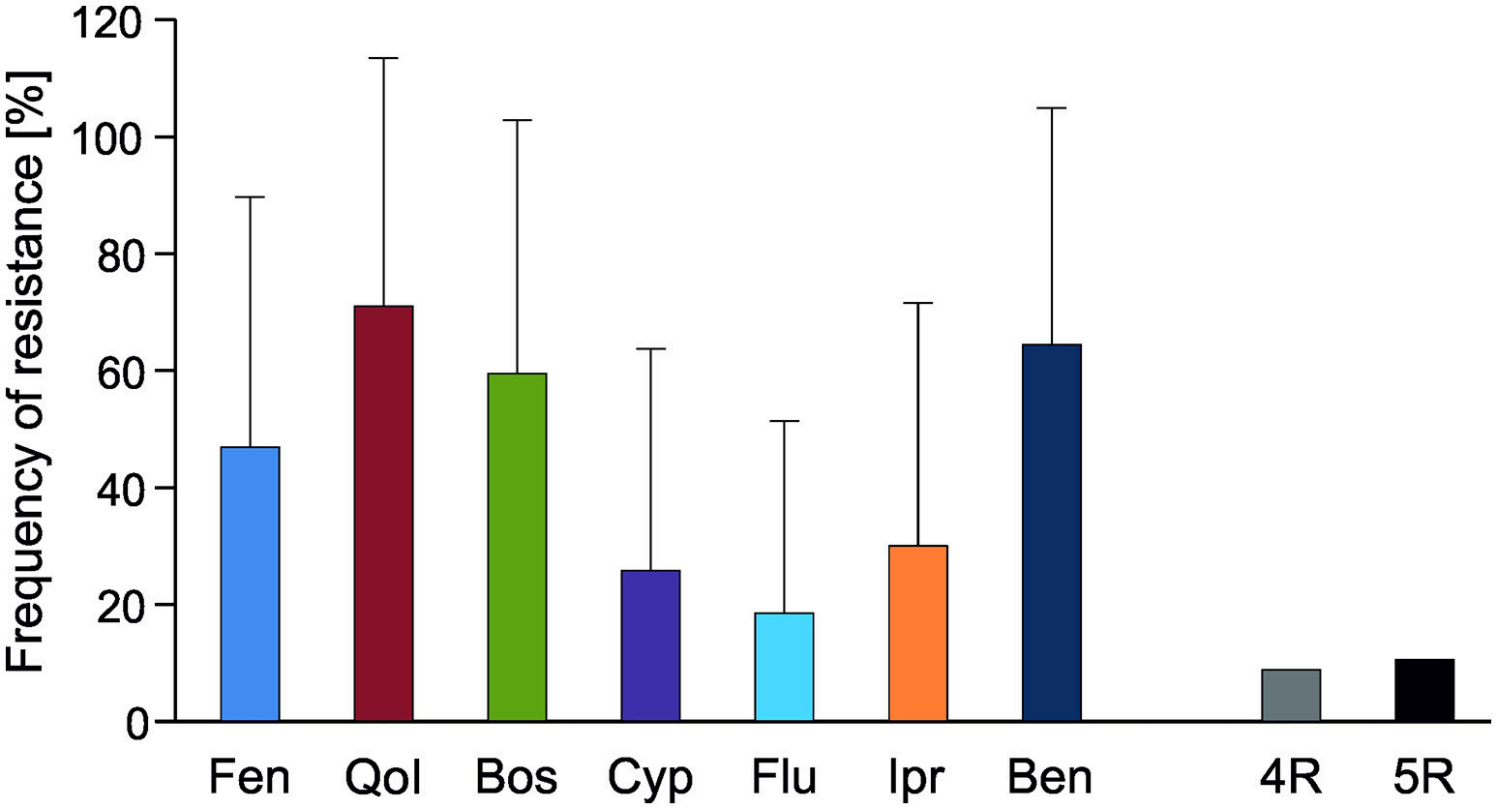
**Average resistance frequencies of ***Botrytis*** isolates from ornamental flowers from 50 sites (cf. Table S2)**. Abbreviations are as in Figure 2.

*Botrytis* isolates from **ornamental flowers** were obtained from greenhouses at 50 different sites in Eastern and Southern Germany. Frequencies of fungicide resistance were highly variable (Table S2). Isolates with 4-fold or 5-fold resistance to current fungicides were recovered from 17 sites. The average fungicide resistance frequencies of flower isolates ranged from 20% against fludioxonil to 75% against QoI (Figure 5). Resistance to benzimidazoles was also very common, even though this fungicide has not been in use for many years. Genetic characterization with species-specific PCR primers (Plesken et al., 2015a) revealed eight isolates to belong to *B. pseudocinerea*; six of them were sensitive to all fungicides and two were resistant only to benzimidazoles. Isolates belonging to *B. cinerea* groups N and S showed a random distribution, with no evidence of any host preference or differences in fungicide resistance frequencies (Table S2). In glasshouses for which no fungicide treatments were reported, MR strains were observed at similar frequencies as in the treated locations (data not shown).

### Genetic and phenotypic characterization of MR strains

For detailed genetic and phenotypic analysis, 19 MR strains (10 of *B. cinerea* group S and 9 of *B. cinerea* group N) were selected from different fields. Most of these strains were also resistant against iprodione and carbendazim (Table 2). Nine MR strains were able to grow on 1 ppm fludioxonil due the ΔL497 deletion mutation in *mrr1* conferring the MDR1h phenotype; the other MR strains grew on 0.2 ppm due to uncharacterized MDR1-related mutations (Leroch et al., 2013). Twenty sensitive strains served as controls, 10 each of *B. cinerea* N and *B. cinerea* S. Analysis of the target genes of the fungicides (except cyprodinil, for which the target site is unknown) revealed common mutations for boscalid (*sdhB*: H272R; H272Y), fenhexamid (*erg27*: F412S, F412I, and T63I), fludioxonil (intermediate resistance due to MDR1h-related mutation ΔL497; Leroch et al., 2013), QoI fungicides (*cytB*: G143A), iprodione (*bos1*: I365N, I365S, and V368F/Q369H) and benzimidazoles (*tubA*: E198A and F200Y) (Hahn, 2014).

**Table 2:** **Resistance mutations and fungicide resistance patterns of multiresistant ***B. cinerea*** strains**.

Strain^*^	**Amino acid substitution**						Resistance^**^						
	***erg27***	***cytB***	***sdhB***	***mrr1***	***bos1***	***tubA***	**Fen**	**QoI**	**Bos**	**Cyp**	**Flu**	**Ipr**	**Car**
MR-S1	F412S	G143A	H272R	ΔL497	V368F/Q369H	E198A	+	+	+	+	±^1^	+	+
MR-S2	F412S	G143A	H272R	ΔL497	I365N	E198A	+	+	+	+	±^1^	+	+
MR-S3	T63I	G143A	H272R	ΔL497	I365N	E198A	+	+	+	+	±^1^	+	+
MR-S4	F412S	G143A	H272R	ΔL497	I365N	E198A	+	+	+	+	±^1^	+	+
MR-S7	F412S	G143A	H272R	ΔL497	I365N	E198A	+	+	+	+	±^1^	+	+
MR-S9	F412S	G143A	H272R	ΔL497	V368F/Q369H	E198A	+	+	+	+	±^1^	+	+
MR-S11	F412S	G143A	H272R	ΔL497	I365S	E198A	+	+	+	+	±^1^	+	+
MR-S14	F412S	G143A	H272R	ΔL497	V368F/Q369H	E198A	+	+	+	+	±^1^	+	+
MR-S19	F412S	G143A	–	n.a.	V368F/Q369H	E198A	+	+	−	+	±^2^	+	+
MR-S29	F412S	G143A	H272R	n.a.	I365N	E198A	+	+	+	+	±^2^	+	+
MR-N1	F412I	G143A	H272Y	–	I365S	−	+	+	+	+	−	+	−
MR-N2	F412I	–	H272Y	n.a.	I365S	−	+	−	+	+	±^2^	+	−
MR-N5	F412S	G143A	H272R	–	–	−	+	+	+	+	−	−	−
MR-N6	F412S	G143A	H272R	–	–	E198A	+	+	+	−	−	−	+
MR-N7	F412S	G143A	H272Y	n.a.	I365N	E198A	+	+	+	+	±^2^	+	+
MR-N8	F412S	G143A	H272Y	n.a.	I365N	E198A	+	+	+	+	±^2^	+	+
MR-N9	F412S	G143A	H272R	–	–	F200Y	+	+	+	−	−	−	+
MR-N10	F412S	G143A	H272R	n.a.	I365N	E198A	+	+	+	+	±^2^	+	+
MR-N11	F412S	G143A	H272R	–	–	−	+	+	+	+	−	−	−

* *Strains MR-Sx belong to B. cinerea group S, strains MR-Nx belong to B. cinerea group N*.

** *Resistance was determined by growth on agar plates containing 5 ppm fenhexamid, 25 ppm azoxystrobin, 7.5 ppm boscalid, 16 ppm cyprodinil, either 0.2 or 1 ppm fludioxonil, 25 ppm iprodione and 5 ppm carbendazim. n.a., not analyzed*.

1 *Growth on 1 ppm fludioxonil (MDR1h)*;

2 *Growth on 0.2 ppm fludioxonil (MDR1)*.

**Figure 6:**
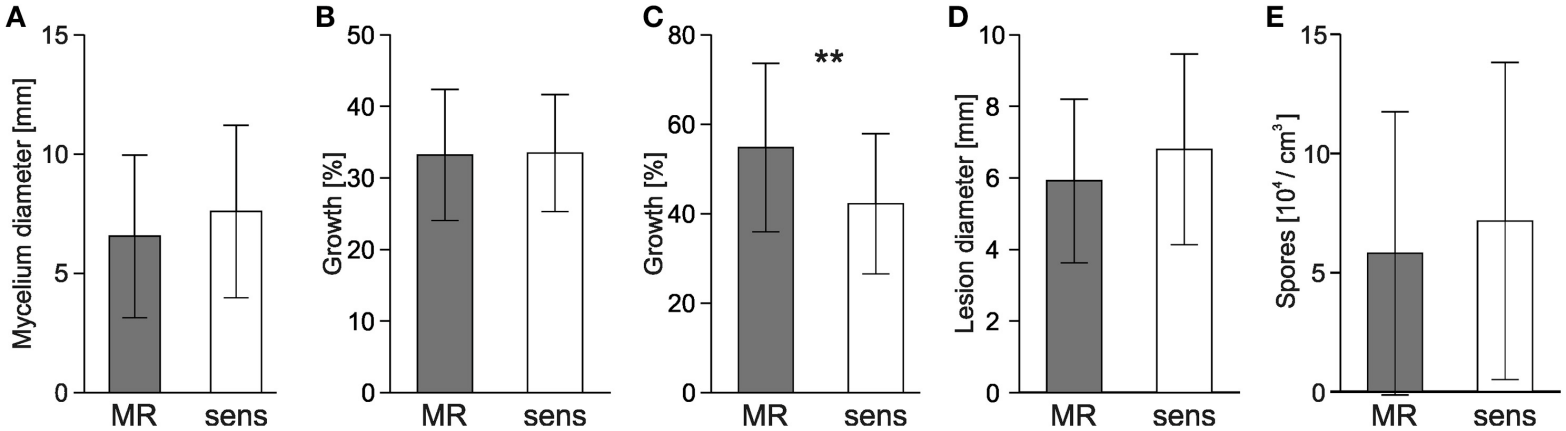
**Growth and infection behavior of ***B. cinerea*** MR and sensitive field strains**. Mean values of 19 MR isolates and 20 sensitive isolates are shown. **(A)** Radial growth on malt extract medium. **(B,C)** Inhibition of radial growth in the presence of 1 M NaCl **(B)** or 10 mM H_2_O_2_ **(C)**. **(D)** Lesion development on detached tomato leaves. **(E)** Sporulation efficiency on malt extract agar. Growth and infection assays **(A–D)** were evaluated after 72 h, sporulation after 14 d. Significant differences at ^**^p < 0.01 are indicated.

For each of the 19 strains shown in Table 2 and for 20 sensitive strains, vegetative growth, sporulation, and infection behavior was compared (Figure 6). Radial growth and sporulation efficiency on HA medium were similar for MR and sensitive strains. In the presence of high salt concentrations (1 M NaCl), growth of MR and sensitive strains was inhibited to a similar extent. Unexpectedly, 10 mM H_2_O_2_ inhibited sensitive strains significantly more strongly than MR strains. Sclerotia were formed by MR and sensitive strains in variable numbers. To compare the viability of sclerotia, two batches of five sclerotia of each of 19 MR strains listed in Table 3, 18 sensitive strains were incubated for 8 months at 10–14°C and then tested for survival. All 370 sclerotia, except one sclerotium from an MR strain, germinated and formed a mycelium. When infection tests were performed on detached tomato leaves, average lesion diameters of MR strains were slightly but significantly smaller than those induced by sensitive strains (Figure 6D). In contrast, lesion formation and sporulation efficiency on inoculated apples were indistinguishable between MR and sensitive strains (Figure 6E).

**Table 3:** **Results of mixed inoculation experiments with MR and sensitive ***B. cinerea*** strains on wounded apple**.

**Mixture**	**Selection**	**Spore germination on selective medium [%]**			
		**Exp. 1**	**Exp. 2**	**Exp. 3**	**Mean**
MR+MDR1: Sensitive	Fen (10 ppm)	74.5	49.8	92.7	72.3±21.5
MR+MDR1h: Sensitive-S	Fen (10 ppm)	100	76.5	63.9	80.1±18.3
MR+MDR1: MR	Flu (0.2 ppm)	100	49.0	24.8	57.9±38.4
MR+MDR1h: MR	Flu (1 ppm)	66.3	58.6	68.0	64.3±5.0

*The following strain mixtures (three strains each) were used: MR+MDR1 (B. cinerea N): MR-N7, MR-N8, MR-N10. MR+MDR1h (B. cinerea S): MR-S4, MR-S9, MR-S14. Sensitive (B. cinerea N): Nsens4, Nsens5, Nsens13. Sensitive-S (B. cinerea S): Ssens1, Ssens2, Ssens3. MR: MR-N1, MR-N5, MR-N11. To each wound, 20 μl of 2 × 10^5^ spores/ml were applied, containing a mixture of six (three plus three) B. cinerea strains*.

### Mixed-inoculation experiments with pairs of sensitive and MR strains of *B. cinerea* on apples

For evaluation of their competitiveness during infection, wounded apples were inoculated with mixtures of three MR and three sensitive strains. We also compared MR strains with and without the MDR1 or MDR1h phenotypes to evaluate the effects of the efflux-based fludioxonil resistance on competitiveness. Conidia recovered from sporulating apple tissue were tested for sensitivity or resistance to a diagnostic fungicide (Table 3). In all experiments, MR strains were at least as competitive as the sensitive strains. Comparison of strain mixtures with and without MDR1 and MDR1h revealed no clear competitive advantage for either group of strains.

### Inoculation experiments to evaluate fungicide efficacy against *Botrytis* MR strains

To estimate the loss of efficacy of fungicides against strains with resistance mutations, inoculation experiments of fungicide-treated host tissues were performed. In a laboratory experiment, detached tomato leaves were treated with fungicides and subsequently inoculated with either a sensitive or an MR strain of *B. cinerea*. While all strains induced large expanding lesions in unsprayed leaf tissue after 72 h (not shown), the sensitive strains were almost completely inhibited by all fungicides even at a 1:10 dilution of the recommended dose. In contrast, the MR strains produced almost normal lesions in the presence of fenhexamid and boscalid, but were strongly inhibited by recommended doses of fludioxonil and cyprodinil. In the presence of cyprodinil and/or fludioxonil at 1:10 dilution, MR strains but not the sensitive strains were able to cause lesions (Table 4).

**Table 4:** Infection of fungicide-treated tomato leaves with sensitive and MR strains of B. cinerea^*^.

	**Frequency of lesion formation [%]**									
	**Fen**		**Bos**		**Flu**		**Cyp**		**Flu**+**Cyp**	
	**Full**	**1:10**	**Full**	**1:10**	**Full**	**1:10**	**Full**	**1:10**	**Full**	**1:10**
MR	83.3 (±37.3)	91.7 (±27.6)	100	100	5.6 (±22.9)	83.3 (±37.3)	5.6 (±22.9)	95.8 (±13.8)	5.6 (±22.9)	91.2 (±27.6)
Sens	0	0	8.3 (±27.6)	8.3 (±27.6)	0	0	0	0	0	0

* *Leaves were sprayed with fungicides at recommended doses (full) and 1:10 dilutions and then inoculated with six MR strains (MR-N5, MR-N10, MR-N11, MR-S3, MR-S4, MR-S7) or five sensitive strains (S-N8, S-N9, S-N11, S-S4, S-S10), each with two or three replications. Infections were rated as successful when expanding lesions were observed after 72 h. Standard deviations are shown in parentheses*.

**Figure 7:**
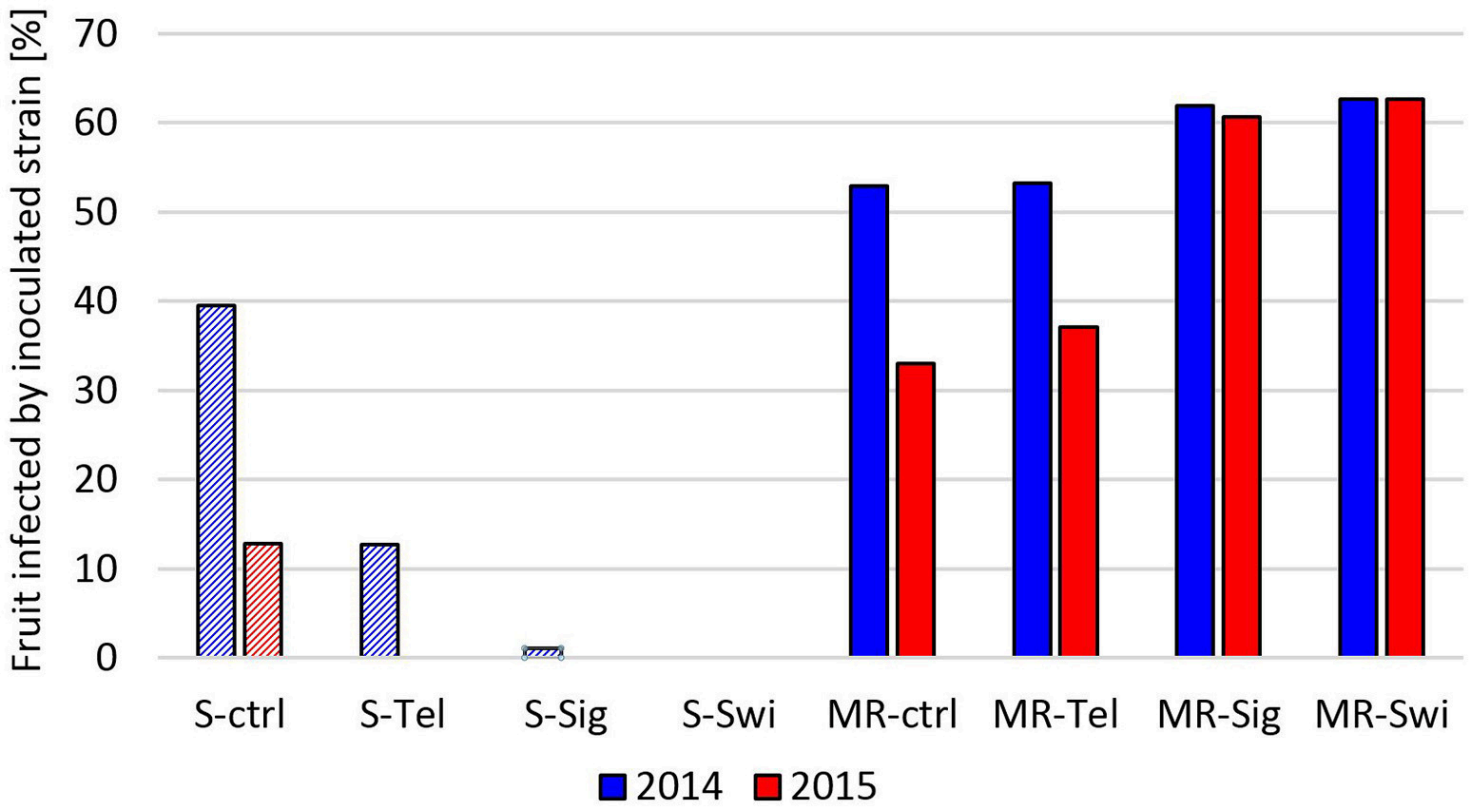
**Infection rate on strawberries grown in the greenhouse (2014) or outdoor (2015) due to inoculation by a sensitive (S, dashed bars) or a multiresistant (MR, filled bars) ***B. cinerea*** strain after treatment with Teldor (Tel; fenhexamid), Signum (Sig; boscalid + pyraclostrobin), Switch (Swi; cyprodinil + fludioxonil), or a water control (ctrl)**.

The efficacy of fungicide treatments against a sensitive and an MR (MDR1h) strain was also tested with potted strawberry plants cultivated in a greenhouse (2014) and outdoors (2015). In these experiments, plants bearing immature fruits were sprayed with fungicides and subsequently inoculated. To identify the strains, conidia isolated from rotting fruit were tested for their fungicide resistance profiles. Strawberries inoculated with the sensitive strain were infected not only by this strain but also by strains with one or more resistances in 59% and 90% of fruits in the 2014 and 2015 experiments, respectively. In contrast, infections of strawberries inoculated with the MR-S4 strain were caused in >80% (2014) and >90% (2015) of the cases by this strain. After subtracting the infections caused by non-inoculated strains, the differential fungicide efficacies against the sensitive and the MR *B. cinerea* strain were evident in both experiments. Treatments with fenhexamid, boscalid + pyraclostrobin or cyprodinil + fludioxonil led to a significant or complete suppression of gray mold rot. In contrast, none of the three fungicides provided any protection against infection by the MR strain in either experiment (Figure 7).

## Discussion

In a survey covering six successive growing seasons we observed high and increasing levels of resistance in *Botrytis cinerea* to all currently used botryticides. These data confirm and extend earlier observations of German *Botrytis* populations from vineyards, strawberries, raspberries and other soft fruits (Leroch et al., 2011, 2013; Weber, 2011). Furthermore, during the past 5 years we have also recorded increasing frequencies of *B. cinerea* strains harboring multiple resistance to most or even all of the currently used fungicides in German horticulture. Similar trends of the accumulation of multiple resistance in *B. cinerea* have been recently reported for strawberries in Greece, the Southeastern USA and China (Amiri et al., 2013; Fernández-Ortuño et al., 2015; Konstantinou et al., 2015; Yin et al., 2015), blueberries in the Western USA (Saito et al., 2016), grapes in Italy (De Miccolis Angelini et al., 2014; Panebianco et al., 2015), tomatoes in Greece and China (Konstantinou et al., 2015; Liu et al., 2016), and ginseng in China (Lu et al., 2016). In this study, we show that multiple resistance is widely distributed in different cultures in Germany, including stone fruits, tree seedlings and ornamental flowers where they have not been reported before. Long-term monitorings in three raspberry fields and a cherry orchard, and comparisons of fields sampled in different years revealed a dramatic increase in the abundance of multiresistant strains on a local as well as national scale. Considering that there were several fields in which resistance frequencies for all fungicides approached 100%, the situation in Germany appears to be at least as dramatic as reports for individual cultures from other countries.

Individual farmers differ strongly in the intensity of their fungicide use, and we have obtained circumstantial evidence that MR strains are most frequent in fields which have been subjected to high fungicide spray intensities for many years (Weber, 2011; Weber and Entrop, 2011). For all strawberry and raspberry fields described here, we know from growers' records that five or more applications of *Botrytis* fungicides were made annually. In a separate study, we have found that fields restricted to three to four applications generally harbor a lower proportion of strains with resistance to individual fungicides as well as multiple resistance, whereas few if any resistant strains are found in untreated plots (Plesken et al., 2015a; Weber, unpublished data). For all other cultures, details of the spraying history were incomplete or lacking altogether, making it impossible to draw correlations between the intensity of treatments and resistance frequencies.

If MR strains of *B. cinerea* are selectively favored by the repeated use of fungicides, how do they reach these intensively treated fields? The first possible explanation is a stepwise accumulation of resistances over time. Our long-term monitoring data in raspberry fields point to this possibility because the first strains with 5-fold multiple resistance were not detected until the third year of our annual survey, but increased in frequency rapidly thereafter. There is evidence that certain strains are particularly competent at developing fungicide resistance because strains with 5-fold resistance to all registered fungicide classes were significantly more likely than non-MR strains to possess resistance to fungicides long phased out, such as benzimidazoles and iprodione (Weber, 2011). Based on similar observations of non-random accumulation of resistances in individual strains, this phenomenon has been proposed to be due to “selection by association” (Hu et al., 2016). A second explanation of the appearance of MR strains is their immigration from other fields. On a transnational scale there is evidence that strains with a special form of efflux-mediated resistance in *B. cinerea* (the so-called MDR2 phenotype) have originated in French vineyards and then migrated to Germany (Mernke et al., 2011). Our present work has provided evidence for the immigration of MR strains into an established cherry orchard and a vineyard. In the latter case, a nearby strawberry field was the likely source, whereas in the case of the cherry orchard the origin of the MR strains remains unclear. A third possibility is the introduction of MR strains into the fields with nursery plants. We have obtained evidence that the canes of raspberry nursery plants often harbor infections by MR strains of *B. cinerea*, and that these can spread to adjacent fields within one growing season (Weber and Entrop, unpublished data). Nurseries over-using the same fungicide classes that are subsequently deployed by the farmers in their production fields could act as efficient propagators of fungicide resistance.

Ornamental flowers in Germany are usually cultivated in greenhouses where the growing conditions, sensitivity to gray mold infection and the frequencies of sprays against *Botrytis* are very diverse. Consequently, strains ranging from completely sensitive to multiresistant were isolated. The abundance of MR strains of *B. cinerea* appeared to be inversely related to the abundance of the sibling species *B. pseudocinerea*, which is unable to develop resistance to most fungicides and is more prominently found in situations where fungicide use is absent or restricted (Plesken et al., 2015a).

Genetic analysis of *B. cinerea* MR strains revealed the commonly found resistance mutations in the target genes of fenhexamid, QoI fungicides, boscalid and cyprodinil. In MR strains of the MDR1h phenotype, the ΔL497 triplet deletion in *mrr1* was found, consistent with previous reports (Leroch et al., 2013; Fernández-Ortuño et al., 2015). There was a high diversity of combinations of resistance mutations in German MR isolates, akin to the situation in the United States (Fernández-Ortuño et al., 2015). These and similar fungicide resistance mutations have sometimes been reported to impair the fitness of *Botrytis* isolates in the case of fenhexamid (Billard et al., 2012), boscalid (Lalève et al., 2013; Veloukas et al., 2014) and iprodione (Leroux et al., 2002) whereas in other studies no fitness reductions were found (Bardas et al., 2008; Fernández-Ortuño et al., 2013; Amiri et al., 2014; Veloukas et al., 2014). The availability of strains with simultaneous resistance to most or all of these fungicides provided the opportunity to analyse their combined effects on fitness by performing phenotypic comparisons of MR and sensitive field strains. Our results from laboratory experiments do not provide evidence of any loss of fitness caused by multiple resistance, including the MDR1(h) phenotype. To the contrary, MR strains appeared even more tolerant to oxidative stress. In mixed inoculation experiments, the MR strains were slightly more competitive than the sensitive strains in the absence of selection. In similar studies, MR strains had shown no differences in growth, sporulation and infection, but were significantly impaired in their competitive ability in the absence of fungicide selection pressure (Fernández-Ortuño et al., 2015; Chen et al., 2016). Owing to the use of different strains, the data from these studies are not directly comparable. However, in general terms we may conclude that *B. cinerea* MR strains have maintained a remarkable fitness, as also indicated by their high abundance in many fields.

Another controversial issue is the effect of fungicide resistance on the performance of the respective fungicides in the field. Our experiments, especially those involving inoculated potted strawberries under conditions approximating the field situation, clearly indicated a nearly complete loss of efficacy of all fungicide groups. The detached-leaf assays confirmed this for fenhexamid, QoI and boscalid, whereas a residual activity was measured for fludioxonil and cyprodinil. This finding correlates with the nature of the efflux-based mechanism underlying the MDR1(h) phenotype of fludioxonil and cyprodinil resistance gives rise to lower resistance factors than most target site mutations (Kretschmer et al., 2009; Hahn, 2014).

Our main conclusions are that MR strains are likely to possess a high fitness in the field and that they are essentially immune to sprays with any of the current botryticides, with the possible exception of the recently released fluopyram (Weber et al., 2015). This has obvious implications for practical horticultural production. For example, MR strains directly undermine any attempt to manage fungicide resistance by alternating between different fungicide classes in successive sprays, as advocated by the fungicide resistance action committee (http://www.frac.info/). To a certain extent, the problem may have been made worse by the registration of fungicide combination products as botryticides. Products featuring a QoI compound are particularly critical, given that QoI resistance due to the G143A mutation is the most widespread of all types of resistance in *Botrytis* (Weber, 2011) and other fungi (Gisi et al., 2002). It is conceivable that the over-use of fungicides against *Botrytis* might actually give a poorer control of gray mold, via the selection of MR strains that are unaffected by any fungicide, than the more modest use of 3–4 sprays at flowering. Fungicide resistance tests should be made available to farmers so that they are able to track the resistance status of the *Botrytis* populations in their fields.

## Author contributions

RW, DR, and PD designed research; SR, DR, PD, and RW performed research; SR, RW, and MH analyzed data; MH and RW wrote the paper.

## Funding

This work was supported by funds of the Federal Ministry of Food and Agriculture based on a decision of the Parliament of the Federal Republic of Germany via the Federal Office for Agriculture and Food under the innovation support program (FKZ 2814705711).

### Conflict of interest statement

The authors declare that the research was conducted in the absence of any commercial or financial relationships that could be construed as a potential conflict of interest.
