# Supplementary material for: Spread of Botrytis cinerea Strains with Multiple Fungicide Resistance in German Horticulture
Source: Front Microbiol. 2017 Jan 3;7:2075. doi: 10.3389/fmicb.2016.02075 (PMC5206850; doi:10.3389/fmicb.2016.02075)
Supplement: Supplementary file 1 [file Table1.docx]

Table S1: Summary of resistance frequencies of *Botrytis* isolates analysed in this study. Resistance frequen­cies for the five currently registered botryticides, i.e. fenhexamid (Fen), QoI compounds, boscalid (Bos), cyprodinil (Cyp) and fludioxonil (Flu), and to former botryticides iprodione (Ipr) and benzimidazoles (Ben) are shown. The occurrence of strains resistant to four (4xR) or all five (5xR) of the registered botryticides is also indicated. Isolates from tree seedlings were from Douglas fir (2014) and heather (2015).

| **Host plant** | **Region-Year** | **Field number**  **(Number of isolates)** | **Resistance frequencies [%]** | | | | | | | **MR frequencies [%]** | |
| --- | --- | --- | --- | --- | --- | --- | --- | --- | --- | --- | --- |
|  |  |  | **Fen** | **QoI** | **Bos** | **Cyp** | **Flu** | **Ipr** | **Ben** | **4xR** | **5xR** |
| Raspberry | North  (2010-2015) [cf. Fig. 1] | 1-2010 (21) | 4 | 38 | 16 | 33 | 6 | 55 | 22 | 0 | 0 |
|  |  | 1-2011 (21) | 24 | 62 | 24 | 52 | 19 | 38 | 19 | 10 | 0 |
|  |  | 1-2012 (27) | 26 | 78 | 52 | 52 | 22 | 52 | 22 | 4 | 15 |
|  |  | 1-2013 (30) | 50 | 83 | 67 | 83 | 57 | 60 | 40 | 23 | 33 |
|  |  | 1-2014 (30) | 77 | 93 | 87 | 80 | 70 | 63 | 60 | 0 | 67 |
|  |  | 1-2015 (30) | 87 | 100 | 100 | 100 | 90 | 90 | 30 | 13 | 87 |
|  | North  (2010-2014) | 2-2010 (29) | 79 | 100 | 3 | 45 | 72 | 100 | 86 | 31 | 0 |
|  |  | 2-2011 (30) | 60 | 97 | 17 | 73 | 70 | 97 | 87 | 37 | 7 |
|  |  | 2-2012 (30) | 57 | 90 | 63 | 53 | 60 | 97 | 90 | 27 | 17 |
|  |  | 2-2013 (30) | 57 | 80 | 83 | 80 | 70 | 93 | 90 | 37 | 27 |
|  |  | 2-2014 (30) | 67 | 73 | 77 | 37 | 50 | 83 | 90 | 20 | 30 |
|  | North  (2010-2015) | 3-2010 (22) | 18 | 14 | 3 | 14 | 18 | 21 | 13 | 18 | 14 |
|  |  | 3-2011 (24) | 15 | 12 | 1 | 19 | 17 | 23 | 17 | 38 | 0 |
|  |  | 3-2012 (23) | 10 | 14 | 10 | 18 | 12 | 15 | 14 | 43 | 4 |
|  |  | 3-2013 (30) | 14 | 24 | 18 | 20 | 20 | 24 | 19 | 33 | 23 |
|  |  | 3-2014 (28) | 8 | 10 | 10 | 20 | 21 | 11 | 24 | 14 | 11 |
|  |  | 3-2015 (30) | 15 | 21 | 22 | 28 | 26 | 18 | 10 | 7 | 50 |
| Strawberry | West-2013 | 1 (21) | 71 | 86 | 76 | 81 | 86 | 76 | 48 | 10 | 62 |
|  |  | 2 (22) | 86 | 73 | 50 | 50 | 91 | 36 | 27 | 5 | 41 |
|  |  | 3 (21) | 81 | 90 | 62 | 71 | 71 | 52 | 38 | 19 | 52 |
|  | South-2013 | 4 (20) | 90 | 100 | 80 | 90 | 95 | 60 | 40 | 20 | 70 |
|  |  | 5 (20) | 100 | 100 | 95 | 100 | 95 | 90 | 55 | 10 | 90 |
|  |  | 6 (20) | 100 | 100 | 65 | 100 | 100 | 75 | 45 | 35 | 65 |
| Strawberry (Everbear) | South-2014  [cf. Fig. 2] | 1a: Treated | 43 | 52 | 41 | 37 | 83 | n.a. | n.a. | 4 | 26 |
|  |  | 1b: Untreated | 76 | 84 | 68 | 73 | 81 | n.a. | n.a. | 30 | 43 |
|  | North-2011 | 2 (18) | 100 | 100 | 72 | 83 | 94 | 94 | 44 | 11 | 72 |
|  |  | 3 (30) | 67 | 80 | 47 | 70 | 57 | 77 | 47 | 30 | 23 |
|  |  | 4 (16) | 100 | 100 | 69 | 100 | 100 | 88 | 44 | 31 | 69 |
|  | North-2015 | 5 (15) | 60 | 100 | 73 | 80 | 100 | 87 | 13 | 7 | 60 |
| Grapevine | Mid-2011 | 1 (31) | 6 | 89 | 4 | 0 | 19 | n.a. | n.a. | 0 | 0 |
| Grapevine | Mid-2014 | 1 (48) | 13 | 77 | 35 | 10 | 13 | 0 | 8 | 0 | 0 |
| Grapevine | South-2014 [cf. Fig. 3] | 1 (16) | 37 | 100 | 72 | 80 | 67 | n.a. | n.a. | 41 | 17 |
| Strawberry |  | 1 (46) | 13 | 88 | 88 | 63 | 69 | n.a. | n.a. | 50 | 13 |
| Cherry | North  (2010-2015) [cf. Fig. 4] | 1-2010 (10) | 10 | 0 | 0 | 0 | 0 | 0 | 0 | 0 | 0 |
|  |  | 1-2011 (15) | 33 | 40 | 20 | 67 | 20 | 27 | 20 | 13 | 13 |
|  |  | 1-2012 (15) | 47 | 47 | 47 | 33 | 20 | 23 | 13 | 0 | 20 |
|  |  | 1-2013 (20) | 25 | 50 | 50 | 20 | 10 | 20 | 30 | 0 | 10 |
|  |  | 1-2014 (13) | 15 | 15 | 15 | 23 | 15 | 23 | 23 | 0 | 15 |
|  |  | 1-2015 (15) | 27 | 87 | 87 | 60 | 27 | 47 | 13 | 7 | 27 |
| Cherry | North-2012 | 1 (15) | 33 | 47 | 47 | 33 | 7 | 13 | 20 | 7 | 7 |
|  | North-2012 | 2 (15) | 40 | 47 | 47 | 33 | 20 | 33 | 13 | 0 | 20 |
|  | North-2013 | 3 (12) | 58 | 83 | 75 | 33 | 8 | 50 | 42 | 0 | 33 |
|  | North-2014 | 4 (18) | 39 | 72 | 61 | 44 | 33 | 56 | 33 | 11 | 28 |
| Plum | North-2012 | 1 (30) | 40 | 80 | 77 | 60 | 43 | 67 | 63 | 3 | 40 |
| Tree seedlings | North | 1-2014 (2) | 100 | 100 | 100 | 100 | 100 | 100 | 0 | 0 | 100 |
|  |  | 2-2015 (2) | 100 | 100 | 100 | 100 | 100 | 100 | 100 | 0 | 100 |
| Flowers | South/East-2015 | 50 (114) | 47 | 71 | 60 | 26 | 18 | 30 | 64 | 9 | 11 |
