# Supplementary material for: Spread of Botrytis cinerea Strains with Multiple Fungicide Resistance in German Horticulture
Source: Front Microbiol. 2017 Jan 3;7:2075. doi: 10.3389/fmicb.2016.02075 (PMC5206850; doi:10.3389/fmicb.2016.02075)
Supplement: Supplementary file 2 [file Table2.docx]

Table S2: Origin, genotype, host plant and fungicide resistance frequencies of *Botrytis* isolates from greenhouse-grown ornamental flowers.

| **Site** | **Region** | **Genotype** | **Host plants** | **Numb. of isolates** | **Number of resistant isolates per site** | | | | | | |
| --- | --- | --- | --- | --- | --- | --- | --- | --- | --- | --- | --- |
|  |  |  |  |  | **Fen** | **QoI** | **Bos** | **Cyp** | **Flu** | **Ipr** | **Carb** |
| 1 | South | BcN | *Lavandula* spp. | 2 | 2 | 2 | 2 | 0 | 0 | 0 | 2 |
| 2 | South | BcN | *Myosotis* spp. | 2 | 1 | 0 | 2 | 0 | 0 | 0 | 1 |
| 3 | South | BcS | *Euphorbia pulcherrima* | 2 | 1 | 0 | 2 | 0 | 0 | 0 | 1 |
| 4 | South | BcN, BcS | *Viola* spp. | 2 | 1 | 0 | 0 | 0 | 0 | 0 | 0 |
| 5 | South | BcN, BcS | *Lysimachia spp., Euphorbia pulcherrima* | 8 | 2 | 4 | 2 | 0 | 2 | 4 | 4 |
| 6 | South | BcN | *Ocimum basilicum* | 2 | 1 | 2 | 1 | 0 | 0 | 0 | 0 |
| 7 | South | BcN, BcS | *Viola* spp. | 2 | 1 | 2 | 1 | 0 | 1 | 0 | 1 |
| 8 | South | BcN, BcS | *Ranunculus* spp., *Leucanthemum* spp. | 3 | 1 | 3 | 3 | 1 | 1 | 2 | 3 |
| 9 | South | BcN | *Euphorbia pulcherrima* | 2 | 1 | 2 | 1 | 1 | 1 | 0 | 2 |
| 10 | South | BcN | *Lavandula* spp. | 1 | 0 | 1 | 1 | 0 | 0 | 0 | 0 |
| 11 | East | BcN, Bps | *Salvia* spp. | 10 | 0 | 8 | 6 | 0 | 0 | 0 | 7 |
| 12 | East | BcN, BcS | *Rosa* spp. | 9 | 1 | 9 | 9 | 3 | 1 | 1 | 7 |
| 13 | South | BcN | *Ranunculus asiaticus* | 2 | 0 | 2 | 1 | 0 | 1 | 1 | 2 |
| 14 | South | BcN, BcS | *Primula vulgaris* | 3 | 3 | 3 | 3 | 2 | 2 | 3 | 1 |
| 15 | South | BcN | *Pelargonium zonale* | 3 | 2 | 3 | 1 | 0 | 0 | 1 | 3 |
| 16 | South | BcN, BcS | *Cyclamen* spp. | 2 | 2 | 2 | 2 | 1 | 1 | 2 | 2 |
| 17 | South | BcN, BcS | *Campanula* spp. | 2 | 1 | 1 | 1 | 1 | 0 | 0 | 1 |
| 18 | South | BcS | *Artocarpus heterophyllus* | 1 | 0 | 0 | 0 | 0 | 0 | 0 | 0 |
| 19 | South | BcN, BcS | *Plantago* spp. | 1 | 1 | 1 | 0 | 0 | 0 | 0 | 1 |
| 20 | South | BcN, BcS | *Pelargonium* spp. | 2 | 0 | 2 | 2 | 1 | 0 | 0 | 1 |
| 21 | South | BcS | *Pelargonium grandiflorum* | 5 | 0 | 5 | 2 | 2 | 0 | 2 | 2 |
| 22 | South | BcN, BcS | *Evolvulus* spp. | 2 | 1 | 1 | 0 | 0 | 0 | 1 | 1 |
| 23 | South | BcN, BcS | *Evolvulus* spp. | 1 | 0 | 1 | 0 | 0 | 0 | 0 | 0 |
| 24 | South | BcS, Bps | *Helleborus* spp. | 2 | 0 | 0 | 0 | 0 | 0 | 0 | 2 |
| 25 | South | BcN | *Muehlenbeckia complexa* | 2 | 1 | 2 | 2 | 1 | 0 | 0 | 2 |
| 26 | South | BcN, BcS | *Primula* spp.*, Ranunculus* spp. | 5 | 1 | 4 | 3 | 1 | 2 | 0 | 3 |
| 27 | East | BcN, BcS | *Pelargonium* spp. | 10 | 9 | 9 | 6 | 5 | 0 | 5 | 9 |
| 28 | East | BcN | *Pelargonium* spp. | 1 | 1 | 1 | 1 | 0 | 0 | 1 | 1 |
| 29 | East | BcS | *Begonia* spp. | 1 | 1 | 1 | 1 | 1 | 1 | 1 | 1 |
| 30 | East | BcN | *Pelargonium* spp. | 1 | 1 | 1 | 0 | 0 | 0 | 0 | 1 |
| 31 | East | BcN | *Nemesia* spp. | 1 | 0 | 1 | 1 | 0 | 0 | 0 | 1 |
| 32 | East | BcS | *Fuchsia* spp. | 2 | 1 | 2 | 1 | 2 | 1 | 1 | 1 |
| 33 | South | BcN | *Lavandula* spp. | 1 | 0 | 1 | 1 | 1 | 0 | 0 | 1 |
| 34 | South | BcS | *Calibrachoa* spp. | 1 | 0 | 1 | 0 | 0 | 0 | 1 | 1 |
| 35 | South | BcS | *Impatiens* spp. | 1 | 1 | 1 | 1 | 1 | 1 | 1 | 1 |
| 36 | South | Bps | *Lavandula* spp. | 1 | 0 | 0 | 0 | 0 | 0 | 0 | 0 |
| 37 | South | Bps | *Pelargonium grandiflorum* | 1 | 0 | 0 | 0 | 0 | 0 | 0 | 0 |
| 38 | South | BcS | *Bacopa* spp. | 1 | 1 | 1 | 1 | 1 | 0 | 0 | 1 |
| 39 | South | BcS | *Begonia* spp. | 1 | 1 | 0 | 1 | 0 | 0 | 0 | 0 |
| 40 | South | BcN | *Lavandula* spp. | 1 | 0 | 0 | 1 | 0 | 0 | 0 | 1 |
| 41 | South | BcN | *Pelargonium peltatum* | 1 | 1 | 1 | 0 | 0 | 0 | 1 | 1 |
| 42 | South | Bps | *Lobellia erinus* | 1 | 0 | 0 | 0 | 0 | 0 | 0 | 0 |
| 43 | South | Bps | *Begonia semperflorens* | 1 | 0 | 0 | 0 | 0 | 0 | 0 | 0 |
| 44 | South | Bps | *Rosa* spp. | 1 | 0 | 0 | 0 | 0 | 0 | 0 | 0 |
| 45 | South | BcS | *Pelargonium* spp. | 2 | 0 | 1 | 2 | 0 | 0 | 0 | 1 |
| 46 | South | BcN | *Pelargonium zonale* | 1 | 1 | 1 | 1 | 1 | 1 | 1 | 1 |
| 47 | South | BcN | *Nemesia* spp. | 1 | 1 | 1 | 1 | 1 | 1 | 1 | 1 |
| 48 | South | BcN | *Pelargonium zonale* | 1 | 1 | 1 | 1 | 0 | 0 | 1 | 1 |
| 49 | South | BcS | *Pelargonium zonale* | 1 | 1 | 1 | 1 | 1 | 1 | 1 | 1 |
| 50 | South | BcN | *Pelargonium zonale* | 1 | 1 | 1 | 1 | 0 | 0 | 0 | 1 |

*Botrytis* genotypes: BcN: *B. cinerea* group N; BcS: *B. cinerea* group S; Bps: *B. pseudocinerea*. Numbers below fungicide abbreviations indicate the numbers of strains with resistance to the respective fungicide.
